# Deep learning based screening and regular assessment of adolescent idiopathic scoliosis using wearable IMU sensors

**DOI:** 10.3389/fbioe.2026.1857499

**Published:** 2026-07-15

**Authors:** Xinyang Tan, Hanwen Lu, Baohong Li, Yi Luo, Fan Feng, Xinyuan Song, Peng Yang, Siyi Zhang, Zhedong Shan, Bosi Li, Zhengxuan Li, Qingyang Jin, Qichao Ma, Quan Li

**Affiliations:** 1 School of Design, Shanghai Jiao Tong University, Shanghai, China; 2 Department of Orthopedics, Shanghai Children’s Hospital, Shanghai, China; 3 Department of Orthopedics, Renji Hospital, Shanghai, China; 4 Carnegie Mellon University, Pittsburgh, PA, United States; 5 Department of Pediatric Orthopedics, Bozhou People’s Hospital, Bozhou, Anhui, China

**Keywords:** adolescent idiopathic scoliosis, Cobb angle, gait, inertial measurement unit, neural network

## Abstract

**Objective:**

The study’s objective is to propose a novel non-invasive method for rapid screening and regular assessment of adolescent idiopathic scoliosis (AIS) through development of a wearable system integrated with multiple inertial measurement units (IMUs) and deep learning models. The system is designed to automatically distinguish between healthy individuals and AIS patients, and subsequently predict the Cobb angle based on continuous temporal kinematic angular sequences acquired during gait.

**Methods:**

Gait kinematic data were acquired from 124 participants (104 patients with average Cobb angle of 21.62 ± 7.93° and 20 healthy subjects) using a 9-IMU wearable device. Extracted angular features were analyzed to quantify bilateral asymmetry, compare differences across severity subgroups, and evaluate their linear correlation with Cobb angles. A two-stage deep learning framework was implemented with a convolutional neural network (CNN) classification model developed for the rapid screening of scoliosis, and a CNN-Transformer model was designed and compared with other five model architectures to predict Cobb angles from the acquired temporal angular sequences.

**Results:**

Scapular kinematics emerged as the most prominent marker of asymmetry, and knee joint kinematics served as the strongest indicator of severity. Meanwhile, angular features of knee, hip and ankle joints demonstrated weak negative linear correlations with Cobb angle. In addition, the scoliosis screening model achieved high predictive performance, with an accuracy of 96.59% and a precision-recall AUC of 0.94 for scoliosis detection. For Cobb angle prediction, the CNN-Transformer model regularized with Gaussian noise during training proved most effective, yielding a mean absolute error of 2.14 ± 0.28° and *R* ^2^ value of 0.85 ± 0.03, outperforming other architectural alternatives evaluated in this study.

**Conclusion:**

Kinematic analysis of angular data validated the efficacy of the wearable system and effectively captured gait characteristics specific to AIS. The deep learning models accurately distinguished scoliosis patients from healthy cases and predicted Cobb angles using temporal kinematic angular sequences, providing a safe, non-invasive, operator-friendly approach suitable for rapid screening and regular assessment.

## 1 Introduction

Adolescent idiopathic scoliosis (AIS) is a complex 3D spinal deformity involving coronal curvature, sagittal malalignment, and axial rotation, leading to postural and kinematic alterations (Wang et al., 2025). Timely detection and regular assessment are essential for management (Schmid et al., 2020), as missed early screening and delayed intervention may lead to curve progression, thereby elevating the risk of surgery, postural deformity, and compromised cardiopulmonary function. The Cobb angle is the standard clinical parameter used to quantify the magnitude of coronal curvature, serving as the primary indicator for assessing severity and guiding treatment decisions (Wang et al., 2018). Although Cobb angle measurement using radiography remains gold-standard, its radiation exposure limits repeated use for frequent assessment. Hence, there exists a compelling clinical need for a non-invasive method for scoliosis screening and periodic assessment of Cobb angle. Although existing screening and assessment methods, for example, scoliometry (Fukuzawa et al., 2025; Liu et al., 2022), standing magnetic resonance imaging (MRI), surface topography (Li et al., 2025; Minotti et al., 2024), time of flight camera (Boché et al., 2025) and thermography (Kwok et al., 2017), are free from X-ray test, they do not directly provide the Cobb angle​ and, moreover, rely heavily on operator expertise or sophisticated instrumentation. Therefore, an approach that integrates operational simplicity with accurate Cobb angle prediction, and extends beyond static postural assessment to enable dynamic kinematic evaluation, is urgently needed.

Inertial measurement units (IMUs) have been extensively employed in kinematic studies (Bogaert et al., 2026; Dal Farra et al., 2025; Villa et al., 2024; Yin et al., 2021), particularly in investigating movement patterns in scoliosis. They are widely used to analyze gait alterations (Veisari et al., 2025), characterize spinal postures (Pelc et al., 2023), examine relationships between gait and spinal alignment (Eun et al., 2024), and assess the impact of bracing in AIS patients (Costantini et al., 2025). For instance, studies attached IMUs to the lumbar spine (Takahashi et al., 2025), embedded them in footwear for group comparisons (Choi et al., 2025), mounted them on the lower back to evaluate trunk rotation (Pau et al., 2018), and arranged multiple sensors along the spine to measure inter-segmental angles during gait (Digo et al., 2020). Although optical motion capture techniques allow precise kinematic analysis (Deng et al., 2024), it requires laboratory settings and complex setup, hindering routine use (Sibson et al., 2024). Therefore, while IMU-based research has advanced understanding of gait and trunk kinematics in AIS, there remains a critical need for practical tools capable of predicting Cobb angle to support rapid screening and frequent assessment.

Recent advances in machine learning have enabled the development of automated Cobb angle measurement systems using X-ray images, which typically employ multi-stage algorithmic architectures incorporating vertebrae identification, vertebral landmark localization (centroids or corners) or spine curve fitting (Alukaev et al., 2022), and subsequent geometric angle calculation (Maeda et al., 2023; Sun et al., 2022; Suri et al., 2023). Studies have been conducted to compare Cobb angles estimated using artificial neural networks and manual measurements (Bernstein et al., 2021; Sun et al., 2022). Retrospective studies were also performed to evaluate the robustness of existing machine learning models (Suri et al., 2023). In addition, classification models were employed to binarily predict whether there is a progression in AIS (Ohyama et al., 2024), to automatically classify severity based on Cobb angle ranges (Yu et al., 2025), and to fast scoliosis detection (Fraiwan et al., 2022). Besides using X-ray images, other studies applied depth sensor data (Kokabu et al., 2021), surface electromyography (Yin et al., 2025) and ultrasonography (Wong et al., 2025) to construct Cobb angle prediction models. Although deep learning models for Cobb angle prediction have been studied, existing approaches mostly rely on X-ray images, limiting their suitability for rapid screening and regular assessment. Few studies have explored Cobb angle prediction based on temporal IMU data to potentially involve kinematics features of AIS. Furthermore, current prediction methods focus on data obtained in static postures, leaving the spatiotemporal motion data for Cobb angle prediction insufficiently explored.

The aim of this study was to develop a novel non-invasive IMU-based method for automatic scoliosis detection and Cobb angle prediction, by developing (1) a nine-IMUs wearable device for rapid exam, and (2) deep learning models combining standardized screening protocol with scoliosis classification and Cobb angle prediction algorithms to enable efficient screening and regular assessment.

## 2 Methods

### 2.1 Participants

This study was approved by the Institutional Review Board for Human Research Protections of Shanghai Jiao Tong University (E20250505I), the Ethics Committee of Shanghai Children’s Hospital (2025R114-E01), and the Ethics Committee of Renji Hospital (KY2024-176-B). Written informed consent was obtained from all participants and their guardians. The inclusion criteria comprised: (1) adolescents aged 6–18 years with clinically diagnosed AIS with Cobb angle of 10–40°, and (2) ability to independently complete gait assessment. Participants were excluded based on the following criteria: (1) presence of neuromuscular disorders; (2) history of spinal surgery; (3) presence of other conditions affecting gait, such as leg-length discrepancy, severe pes planus, fractures or arthritis; (4) having significant spinal pain; and (5) diagnosis of non-AIS spinal pathologies. Totally 104 patients and 20 healthy participants were recruited. Their demographic information is shown in Table 1.

**TABLE 1:** Demographics of study participants.

Variable	AIS group (n = 104)	Healthy controls (n = 20)	p
Age (years), mean ± SD	13.51 ± 2.18	13.00 ± 2.62	0.442
Sex, (Female)/(Male)	89/15	14/6	0.106
BMI (kg/m2), mean ± SD	17.83 ± 2.70	17.63 ± 2.41	0.773
Cobb angle (°), mean ± SD	21.62 ± 7.93	N/A	N/A
Cobb angle severity, n (%)			
Mild (10° ≤ Cobb <30°)	84 (80.8%)	N/A	N/A
Moderate (30° ≤ Cobb ≤40°)	20 (19.2%)	N/A	N/A
Main curve direction, n (%)			
Right	50 (48.1%)	N/A	N/A
Left	54 (51.9%)	N/A	N/A

Abbreviations: AIS, adolescent idiopathic scoliosis; SD, standard deviation; BMI, body mass index; N/A, not available.

### 2.2 IMU-based wearable device

**FIGURE 1:**
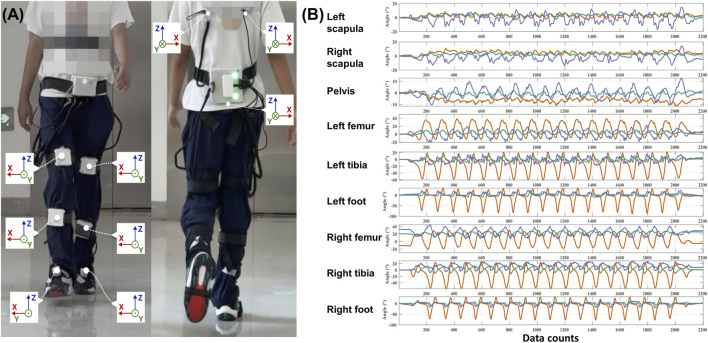
**(A)** Schematic illustration of the wearable IMU placement for capturing temporal angular sequences during gait. Written informed consent was obtained from all participants and their guardians. **(B)** Raw continuous angular data at critical anatomical locations.

As shown in Figure 1, an IMU-based wearable system was utilized, featuring one sacrum-mounted IMU for pelvic kinematics and six on lower limbs for hip, knee and ankle motion. Two IMUs were placed over the central third of the scapular spine between the angulus acromialis and the trigonum spinae, following established IMU placement protocols for scapular kinematics (Antonacci et al., 2023). This region overlies the bony ridge directly, where soft tissue coverage is minimal. The system recorded quaternion-orientation data at 100 Hz onto an SD card. The design utilized elastic wiring and adjustable straps for securing sensor placement during gait, allowing application and removal of the wearable device under 3 min. The device is lightweight (500 g) without exerting excessive pressure or mechanical tension that could interfere with natural movements.

### 2.3 Experiment

Participants wearing the device were instructed to walk at a self-selected comfortable speed for about 14 complete gait cycles. Prior to gait data collection, an initialization procedure was conducted to compensate for the arbitrary mounting orientation of each IMU unit. As each sensor reported orientation in its own coordinate frame, raw output varied with placement. To establish a consistent anatomical reference, the sensors were powered on for at least 5 min to allow filter stabilization. Participants were then asked to maintain a standardized neutral upright posture. An alignment reset was applied to all nine IMU channels, zeroing the roll, pitch, and yaw readings. The reset was repeated at the start of each trial, ensuring consistent referencing across sessions. Participants completed at least four experimental trials, with intervals between trials, to ensure the acquisition of qualified signals for subsequent analysis. In addition, the Cobb angles of the primary curve for all patients were manually measured on full-spine radiographs by experienced clinicians. Following standard protocol, each measurement identified the superior and inferior end vertebrae of the curve, and the angle was defined by the intersection of lines drawn along their respective endplates. To enhance reliability, the process was performed independently by two clinicians, and the average of their measurements was recorded as the final Cobb angle.

### 2.4 Data processing and statistical analysis

IMU signals were processed using an onboard Kalman-based sensor fusion algorithm that fuses gyroscope, accelerometer, and magnetometer data in real time, minimizing cumulative output drift. Each angular channel was subsequently processed by a 5-point median filter to suppress impulsive spike artifacts (Hori et al., 2020), followed by a 4th-order zero-phase Butterworth low-pass filter at an 8 Hz cut-off to remove high-frequency noise. Analysis focused on ten steady-state gait cycles to avoid initial and terminal gait effects. To empirically verify that no meaningful drift accumulated over the 14 recorded cycles, cycle-to-cycle consistency was quantified.​ Specifically, joint angles at the right-toe-off event from 14 consecutive gait cycles were extracted for the pelvis, scapula, and knee pitch channels. Averaged across 124 subjects, the average cycle-to-cycle root mean squared error (RMSE) was 0.70 ± 0.27° for the pelvis, 0.92 ± 0.64° for the scapula, and 0.98 ± 0.46° for the knee. These small average RMSE values confirmed that the angular sequences remained stable across cycles, indicating that any residual drift was negligible for subsequent analysis. Moreover, knee, ankle, hip, pelvic, and scapular rotational angles (9 variables in three axes) were derived from quaternion data, uniformly resampled to 1200 points.

Statistical analyses were conducted to validate the IMU-based wearable system’s capacity to capture kinematic features associated with AIS. First, bilateral asymmetry (left vs. right) was assessed from the angular features (i.e., mean, standard deviation (SD) and range of motion (ROM)) to detect asymmetric patterns in targeted joints of AIS patients and confirm their consistency with prior literature, thereby verifying appropriate sensor placement. Second, angular features were compared across severity groups (non-scoliosis, 10°≤Cobb<30° and 30°≤Cobb≤40°, according to literature (Yu et al., 2025)) to evaluate the system’s ability to discriminate between healthy controls and AIS patients, as well as between mild and moderate subgroups. Third, linear regression was performed to examine correlations between IMU-derived angular features and Cobb angles, assessing the presence of a linear relationship. All p-values were adjusted using the Benjamini–Hochberg false discovery rate (FDR) procedure. These analyses served two purposes: (1) to determine the feasibility of extracting AIS-related information from the IMU-based wearable device, and (2) to evaluate the adequacy of linear correlations between kinematic features and Cobb angles, and consequently, the need for nonlinear models to improve group discrimination and Cobb angle prediction.

### 2.5 Scoliosis screening model

A scoliosis screening model was developed using the convolutional and fully connected neural network (CNN-FNN) architecture to perform classification between non-scoliosis participants (Cobb<10°) and scoliosis patients (10°≤Cobb<40°) from temporal angular sequences of participants’ movements. A total of 469 samples were utilized, each comprising a 1,200 × 3 × 9 data array representing sequential angular measurements across 9 channels and 3 spatial axes. Throughout model development and evaluation, to ensure a clinically realistic evaluation, the dataset was divided using a patient-wise split with 80% of patients allocated for model training and the remaining 20% held out as a test set. The hyperparameters were optimized through iterative trials, and the training employed an early stopping criterion, halting when the validation loss showed no improvement over 50 consecutive epochs. The model’s architecture comprised three one-dimensional (1D) convolutional (Conv) layers, each configured with kernel sizes (5, 5 and 5), a padding of 2, a uniform stride of 1, and progressively increasing filter numbers (32, 64 and 128), followed by max pooling with window size of 2 and stride of 1. Three fully connected (FC) layers with hidden units (128, 64, and 32) were constructed, followed by a dropout layer with a rate of 0.3. A softmax output was utilized for classification. The learning rate was set as 1e-4. All Conv and FC layers employed rectified linear unit (ReLU) activation function.

### 2.6 Cobb angle prediction model

**FIGURE 2:**
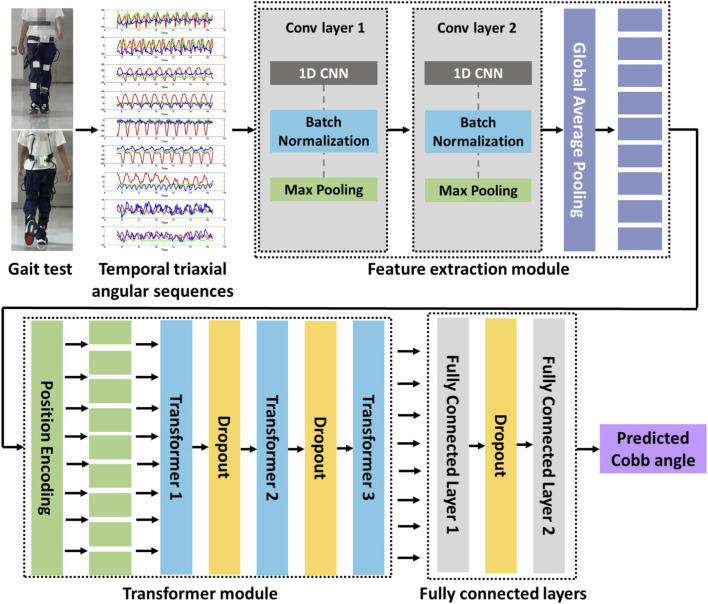
Model architecture of the Cobb angle prediction model.

A deep neural network was developed for Cobb angle prediction in scoliosis utilizing 358 patient samples. The model accepts triaxial temporal angular sequences as input and outputs a corresponding Cobb angle value. As shown in Figure 2, the model architecture integrates two core modules: a feature extraction module and a Transformer module. The feature extraction module employs two 1D convolutional layers, each followed by max pooling (kernel size = 2), to extract local spatiotemporal features. These are then condensed via global average pooling. Subsequently, the Transformer module, with its multi-head self-attention mechanism, captures long-range dependencies within the sequential kinematic data. The Transformer module comprises three layers, each with four multi-head attention mechanisms, interspersed with dropout layers (rate of 0.2) for regularization, and culminates in a linear output layer for regression.

The development of the Cobb angle prediction model employed an 8:2 split of patients’ dataset for training and internal testing, respectively. A 5-fold cross-validation scheme was rigorously applied within the training set for hyperparameter optimization, with the final configurations detailed in Table 2. To mitigate overfitting during the five-fold training process, Gaussian noise (mean = 0, standard deviation = 0.05) was added to the sequential angular data as a data augmentation strategy to enhance the model’s robustness to minor input perturbations and reduce overfitting to local details or noise patterns in the training samples. This approach was adopted based on the performance of the model validation set and the literature (Salah Al-Shati and Mohammed, 2026). Moreover, the Huber loss function was adopted for model training to enhance robustness to potential outliers, coupled with an early stopping criterion that terminated training if the validation loss stabilized over 300 consecutive epochs.

**TABLE 2:** Hyperparameters of the Cobb angle prediction model.

Structure	Conv layers	Kernel size	Padding	Out-channels	Transformer		FC	Dropout	LR	L2 regulari-zation
					*d*_*model*_	*d*_*feedforward*_	​	​	​	​
Hyper-parameter	2	(61,61)	(30,30)	(8,16)	16	64	(144,32)	0.2	5e-5	1e-5

Abbreviations: d_model_: Feature dimension; d_feedforward_: Feed-forward hidden dimension; FC: fully connected layer; LR: learning rate.

An independent external test set was formed by randomly selecting and withholding 11 patients from the main dataset, nine from the 10–30° subgroup and two from the 30–40° subgroup. In addition to the 5-fold cross-validation, this small external hold-out test set provides a supplementary, stringent and unbiased assessment of the model’s generalizability to entirely unseen individuals (Hassan et al., 2025). Besides, four alternative deep neural network architectures, including CNN + Transformer (identical to the proposed model but without Gaussian noise), CNN + LSTM (Long Short-Term Memory), CNN + GRU (Gated Recurrent Unit), CNN + FC and Random Forest, were constructed and their performances were compared. The hyperparameters of the alternative models are presented in the Supplementary Table S1. All training and test experiments were implemented on PyTorch with one NVIDIA RTX 5060 GPU.

## 3 Results

### 3.1 Angular features between left and right sides in AIS

**FIGURE 3:**
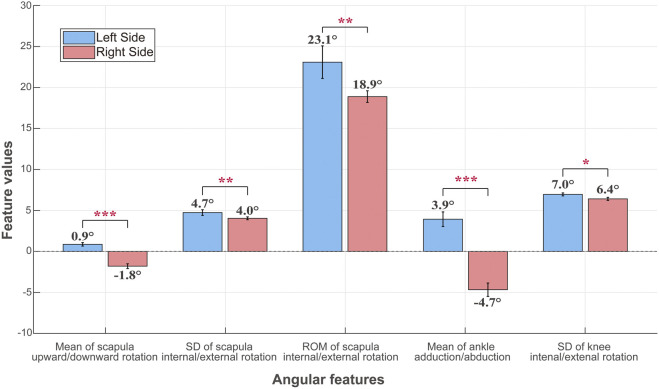
Bilateral asymmetries across ankle, knee, and scapular kinematic features. **: p < 0.01; ***: p < 0.001.

The paired T-tests and Wilcoxon signed-rank tests demonstrated pronounced bilateral asymmetries across ankle, knee and scapular kinematics (Figure 3). Scapular kinematics exhibited marked asymmetry in upward/downward rotation mean (absolute average difference (AD) = 2.66°, p < 0.001), and internal/external rotation in both SD (0.70°, p < 0.01) and ROM (AD = 4.20°, p < 0.01). Kinematic asymmetry was also revealed a significant difference in angular mean of ankle adduction/abduction (AD = 8.60°, p < 0.001). Knee analysis revealed substantial difference in internal/external rotation SD (AD = 0.54°, p < 0.05).

### 3.2 Angle features among severity groups

**FIGURE 4:**
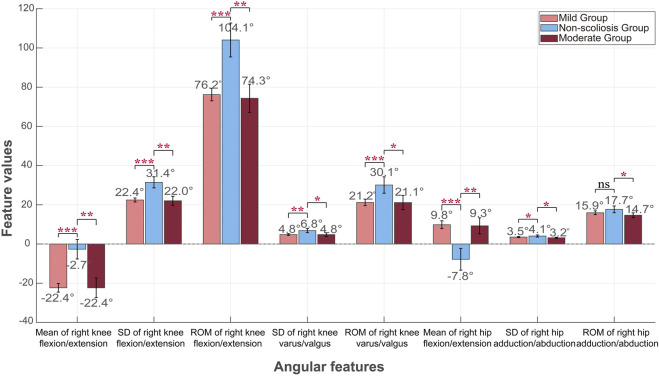
Significant differences in knee and hip angular kinematic features among non-scoliotic, mild and moderate scoliosis groups. *: p < 0.05; **: p < 0.01; ***: p < 0.001.

Kruskal–Wallis tests identified significant differences in knee and hip angular kinematics between non-scoliotic and scoliotic groups (Figure 4). Mann-Whitney U tests revealed that, relative to healthy controls, the mild group exhibited significant alterations in right knee flexion/extension, specifically in mean (AD = 19.69°, p < 0.001), SD (AD = 9.00°, p < 0.001), and ROM (AD = 27.86°, p < 0.001), as well as in right knee varus/valgus ROM (AD = 8.91°, p < 0.001) and SD (AD = 2.02°, p < 0.01). The mild group also differed significantly in right hip flexion/extension mean (AD = 17.64°, p < 0.001) and right hip adduction/abduction SD (AD = 0.54°, p < 0.05). More extensive differences were observed between non-scoliosis and moderate group, including larger effect on the right knee flexion/extension (mean: AD = 19.71°, p < 0.01; SD: AD = 9.41°, p < 0.01; ROM: AD = 29.75°, p < 0.01), varus/valgus (ROM: AD = 8.96°, p < 0.05; SD: AD = 2.04°, p < 0.05), right hip flexion/extension mean (AD = 17.08°, p < 0.01), and right hip adduction/abduction (SD: AD = 0.90°, p < 0.05; ROM: AD = 3.00°, p < 0.05).

### 3.3 Linear correlation between angle features and Cobb angle

**FIGURE 5:**
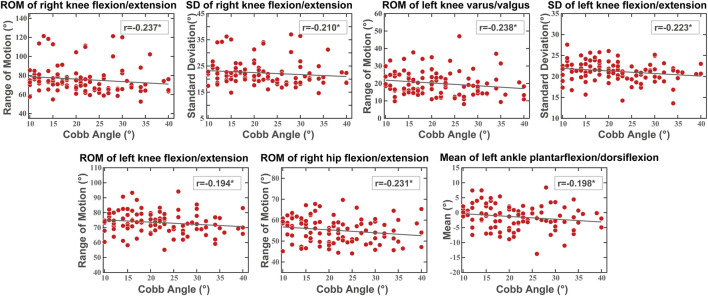
Linear correlations between angular kinematic features and Cobb angle.

As illustrated in Figure 5, Spearman correlation analysis identified seven angular features significantly correlated with Cobb angle, especially at knee joint (p < 0.05). Weak negative associations were observed for right knee flexion/extension (ROM: r = −0.24; SD: r = −0.21), left knee varus/valgus (ROM: r = −0.24) and flexion/extension (SD: r = −0.22; ROM: r = −0.19), right hip flexion/extension (ROM: r = −0.23), and left ankle plantar flexion/dorsiflexion mean (r = −0.20).

### 3.4 Performance of the scoliosis screening model

**FIGURE 6:**
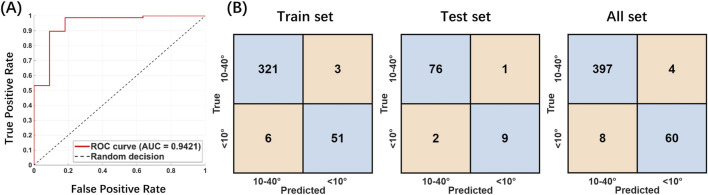
Scoliosis screening model evaluation results. **(A)** ROC curve of the scoliosis screening model. **(B)** Confusion matrices for the training set, test set, and the entire cohort of the scoliosis screening model.

The proposed classification model achieved high performance, with an accuracy of 96.59%, a sensitivity of 98.70% and an area under the receiver operating characteristic curve (AUC) of 0.94, indicating excellent discriminative capability between healthy controls and patients with scoliosis. The corresponding receiver operating characteristic (ROC) curve of the deep learning model for scoliosis screening is presented in Figure 6A. Detailed confusion matrices for the training set, test set, and the entire cohort are provided in Figure 6B, with comprehensive performance metrics summarized in Table 3. These results demonstrate the model’s strong ability to reliably detect scoliosis based on temporal angular sequences of key body segments during gait, underscoring its potential as a non-invasive, portable tool for rapid clinical screening.

**TABLE 3:** Evaluation parameters of the scoliosis screening model.

Dataset	Accuracy	Precision	Recall/Sensitivity	F1 score	Specificity	AUC
Training	97.64%	98.17%	99.07%	98.62%	89.47%	0.9933
Test	96.59%	97.44%	98.70%	98.06%	81.82%	0.9421
All	97.44%	98.02%	99.00%	98.51%	88.24%	0.9860

Abbreviations: AUC, the area under the receiver operating characteristic curve.

### 3.5 Performance of Cobb angle prediction models

**FIGURE 7:**
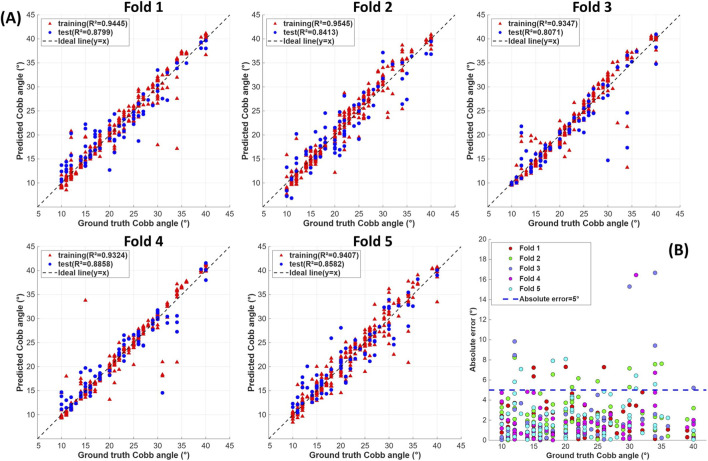
Cobb angle prediction model evaluation results. **(A)** Linear fitting between the predicted Cobb angles and ground truth Cobb angles in 5-fold cross-validation. **(B)** Absolute errors of the predicted Cobb angles.

The predictive performance of the evaluated models is summarized in Table 4. The proposed architecture (CNN-Transformer regularized with Gaussian noise) achieved the lowest mean absolute error (MAE) of the testing set (2.14 ± 0.28°) and the highest *R* ^2^ (0.85 ± 0.03) in 5-fold cross-validation, demonstrating its superiority over all alternative models. The strong agreement between predicted and ground-truth Cobb angles for both the training and test sets in 5-fold cross-validation is visualized in the scatter plots of Figure 7A, with overall predicted absolute errors illustrated in Figure 7B.

**TABLE 4:** Evaluation parameters of the Cobb angle prediction models.

Models	Training				Test				External test			
	MAE	MSE	RMSE	*R* ^2^	MAE	MSE	RMSE	*R* ^2^	MAE	MSE	RMSE	*R* ^2^
CNN+ Transfor-mer (noise)	1.27 ± 0.14	4.28 ± 0.48	2.07 ± 0.11	0.94 ± 0.009	2.14 ± 0.28	9.59 ± 2.08	3.08 ± 0.32	0.85 ± 0.03	2.75	10.24	3.20	0.73
CNN+ Transfor-mer	1.10 ± 0.15	3.24 ± 0.50	1.79 ± 0.14	0.95 ± 0.008	2.31 ± 0.20	12.03 ± 1.81	3.46 ± 0.25	0.82 ± 0.03	2.77	11.30	3.36	0.71
CNN+ LSTM	1.73 ± 0.15	10.01 ± 2.96	3.13 ± 0.48	0.84 ± 0.04	3.29 ± 0.40	24.44 ± 8.41	4.87 ± 0.85	0.62 ± 0.14	3.50	18.44	4.29	0.51
CNN+ GRU	1.62 ± 0.17	5.53 ± 0.96	2.34 ± 0.21	0.91 ± 0.01	3.09 ± 0.26	16.97 ± 3.54	4.10 ± 0.44	0.74 ± 0.05	3.35	20.33	4.51	0.45
CNN+ FC	1.96 ± 0.33	10.93 ± 6.06	3.19 ± 0.86	0.83 ± 0.09	3.49 ± 0.52	30.47 ± 15.22	5.35 ± 1.37	0.52 ± 0.24	2.53	12.91	3.59	0.65
Random forest	2.35 ± 0.07	9.03 ± 0.37	3.00 ± 0.06	0.86 ± 0.005	4.98 ± 0.39	39.10 ± 6.62	6.23 ± 0.51	0.39 ± 0.09	4.44	29.93	5.47	0.31

Abbreviations: MAE, mean absolute error; MSE, mean squared error; RMSE, root mean squared error; *R* ^2^, R-squared value.

A detailed comparison of alternative architectures revealed a clear performance hierarchy. The predicted and ground-truth Cobb angles for both the training and testing sets in 5-fold cross-validation of the alternative models are visualized in the scatter plots in the Supplementary Figure S1. The standard CNN-Transformer model without added noise yielded satisfactory results with MAE of the testing set = 2.31 ± 0.20° (*R* ^2^ = 0.82 ± 0.03), indicating that the introduced Gaussian noise served as an effective regularizer, effectively reducing overfitting and improving generalization. The CNN-LSTM, CNN-GRU and CNN-FC models showed moderate performance with MAE of testing set = 3.29 ± 0.40° (*R* ^2^ = 0.62 ± 0.14), 3.09 ± 0.26° (*R* ^2^ = 0.73 ± 0.05) and 3.49 ± 0.52° (*R* ^2^ = 0.52 ± 0.24), respectively, while the Random Forest model exhibited the weakest performance in testing set (MAE = 4.98 ± 0.39°, *R* ^2^ = 0.39 ± 0.09).

**FIGURE 8:**
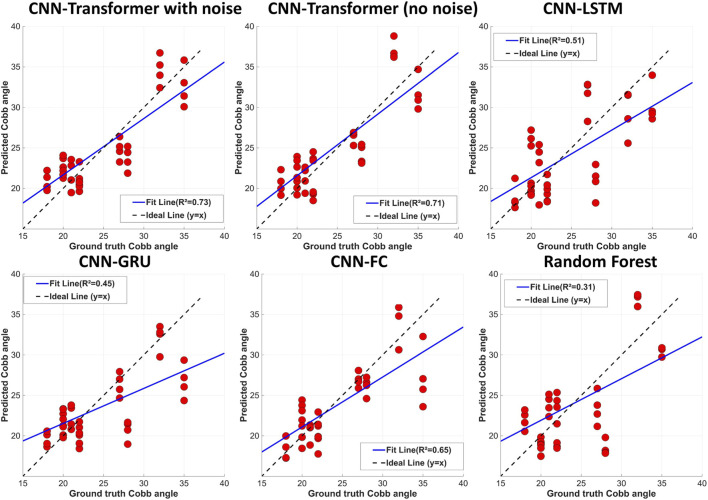
Linear fitting between predicted and ground-truth Cobb angles of all models in the external tests.

The generalizability of the proposed model was additionally validated on a completely independent external test set comprising 11 unseen individuals. The predicted and ground-truth Cobb angles for the external test sets of all models are presented in Figure 8. In this supplementary assessment, the CNN-Transformer model with Gaussian noise also delivered the optimal predictive performance with low MAE (2.75°), lowest RMSE (3.2°) and highest *R* ^2^ (0.73). The standard CNN-Transformer followed closely (MAE = 2.77°, *R* ^2^ = 0.71), while the Random Forest model presented relatively lower accuracy (MAE = 4.44°, *R* ^2^ = 0.31). These results collectively establish the CNN-Transformer architecture augmented with Gaussian noise as the most effective and generalizable model for IMU-based Cobb angle prediction.

## 4 Discussion

Kinematic analysis revealed significant bilateral asymmetries in scapular, ankle and knee angular features during gait among AIS patients. The most pronounced asymmetries were observed in scapular angular kinematics, aligning with existing literature (Fukuzawa et al., 2025; Han et al., 2023). It particularly occurred in upward/downward rotation and internal/external rotation, likely resulting from muscular imbalances between the serratus anterior and trapezius muscles. These muscles normally act synergistically to maintain dynamic scapular stability. However, their disrupted coordination alters scapulohumeral rhythm and leads to observable kinematic deviations. Further asymmetries were detected in ankle adduction/abduction, indicating adaptive foot-ground strategies to maintain balance, and in knee internal/external rotation, reflecting reduced rotational consistency.

Statistical analyses revealed significant kinematic differences between non-scoliosis controls and AIS patients. Specifically, reduced ROM in knee varus/valgus and flexion/extension were observed in AIS group, consistent with existing literature (Mahaudens et al., 2009), which may indicate reduced knee joint compliance during gait. Hip joint kinematics further distinguished groups, with patients exhibiting decreased ROM in hip adduction/abduction and elevated joint angles in hip flexion/extension, presumably due to compensatory pelvic positioning strategies to maintain balance. Furthermore, following FDR correction, no significant differences were identified between the mild and moderate AIS groups. This may imply that substantial information for further distinguishing severities could lie in nonlinear interactions and temporal dependencies which could not be directly reflected by linear analyses, thereby underscoring the potential utility of non-linear modelling for more nuanced prediction.

In addition, significant linear correlations were identified between Cobb angle and specific angular features of knee and hip joints, consistent with prior research (Seo et al., 2013). However, all significant correlations were mild, indicating that univariate linear relationships are insufficient to explain Cobb angle variation, thereby necessitating the development of nonlinear models to better capture the complex interplay between kinematic angular features and Cobb angle.

The scoliosis screening model demonstrated high classification performance, achieving an accuracy of 96.59% in distinguishing between non-scoliotic and scoliosis groups. This performance compares favorably with existing machine learning-based scoliosis classification models, which report accuracies ranging from 73.3% to 86% using X-ray images (Yu et al., 2025; Zhang et al., 2023) and 90% using surface topography data (Rothstock et al., 2020). Samadi et al. achieved 91.4% accuracy using an ensemble voting classifier trained on optokinetic data from 26 landmarks combined with treadmill-mounted force sensor measurements (Samadi et al., 2023). Notably, Cho et al. also utilized IMU data for scoliosis severity classification, achieving an accuracy of 85.7% using a support vector machine (Cho et al., 2018). However, their study relied exclusively on extracted gait features rather than more comprehensive temporal angle sequences, which may contain richer kinematic information reflective of spine deformity. Furthermore, compared with their methods, the proposed model incorporated scapular motions, which may be a significant biomechanical indicator of scoliosis kinematics. Although large language models such as PMC-LLaMa13B and ChatGPT4 (73%–100%) demonstrated competitive accuracy using X-ray images (Fabijan et al., 2023; 2024), our proposed method offers a substantial practical advantage by achieving comparable performance with significantly reduced data requirements and an offline, radiation-free measurement process suitable for rapid clinical deployment.

The CNN-Transformer architecture augmented with Gaussian noise was identified as the optimal model for predicting Cobb angles from temporal angular sequences. The model achieved a MAE of 2.14° ± 0.28° on the internal test set and 2.75° on a supplementary independent external set. These errors are well within the reported variability of manual Cobb angle measurement from full-spine radiographs, which is typically considered to be approximately 5° (Ricart et al., 2011). It should be noted that, the MAE of 2.14° achieved by our model should be interpreted in the context of clinical decision thresholds for AIS management. According to established guidelines, key Cobb angle thresholds include 10° (diagnostic threshold for AIS), 10–40° (initiation of bracing and monitoring of curve progression), and >40° (consideration for surgical intervention) (Anthony et al., 2021; Bettany-Saltikov et al., 2015). The MAE is substantially smaller than these clinically meaningful intervals, suggesting that the model’s prediction uncertainty is unlikely to cause systematic misclassification across major treatment boundaries. However, caution should be warranted for borderline cases near critical thresholds, where a prediction error of ∼2° could theoretically shift a patient across a decision boundary. In such scenarios, our model is intended as a screening and assessment tool with confirmation via radiography recommended before treatment decisions are made. Overall, this level of accuracy supports the model’s utility for frequent, non-invasive screening and assessment, while maintaining the gold-standard X-ray for definitive surgical or bracing decisions. In addition, the external test set did not serve as the sole or primary basis for model performance evaluation in this study, but rather supplemented the results of the 5-fold cross-validation to additionally verify its generalization. Although it only included 11 patients, their Cobb angles covered varying degrees of severity within the clinically relevant range of 10°–40°, indicating that this supplementary external test set was not limited to a narrow Cobb angle level.

**FIGURE 9:**
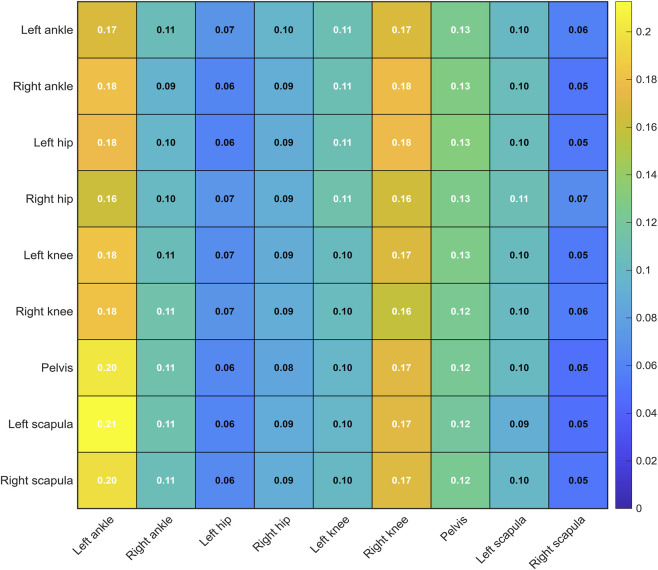
Self-attention weights of the Transformer module.

As shown in Figure 9, the self-attention weights of the Transformer module indicated that the model assigned relatively higher attention weights to features related to the left ankle and right knee, suggesting that kinematic information from these joints played a more important role in the model’s prediction. Additionally, kinematic analysis also revealed significant bilateral asymmetries in ankle and knee angular features during gait. This consistency indicates that the model did not rely on arbitrary patterns, but learned joint-specific movement information relevant to Cobb angle estimation. Moreover, this attention-based finding is also consistent with our statistical analysis. As shown in Figure 4, among the eight statistically significant kinematic features, five were derived from the right knee. This consistency suggests that right knee kinematics were not only associated with Cobb angle in the statistical analysis, but were also emphasized by the Transformer model during prediction.

The comparative analysis of model architectures revealed that the Transformer module with self-attention mechanism was superior to LSTM, GRU, fully-connected networks and Random Forest in capturing long-range dependencies and richer kinematic features from the temporal angular sequences. Moreover, the deliberate addition of Gaussian noise during training enhanced the model’s robustness to the inherent variability and minor artifacts in real-world IMU signals, thereby improving its generalization performance on unseen patient data. Based on the method proposed in this study, future research will test different levels of noise during training to further improve model robustness and generalization across different populations, devices, and clinical environments. In comparison to recent automated Cobb angle estimation methods based on X-ray images which reported MAEs of 0.5°–2.51° (Hayashi et al., 2024; Suri et al., 2023; Wang et al., 2024), the proposed model integrated with an IMU-based wearable system achieved comparable accuracy while offering a safe, non-invasive, operator-friendly alternative suitable for rapid screening and regular assessment.

## 5 Potential clinical application

**FIGURE 10:**
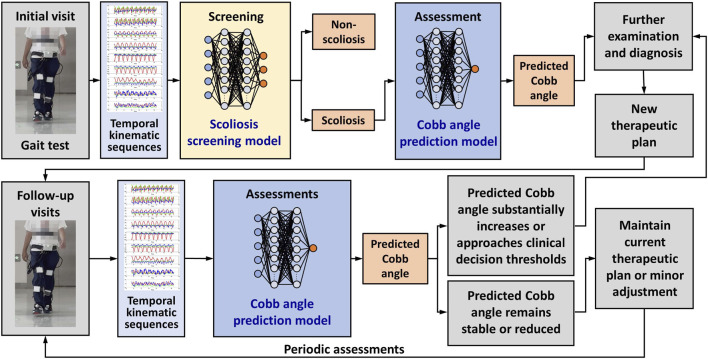
The potential application of the proposed method for rapid, non-invasive scoliosis screening and regular assessment of AIS in clinical settings.

Figure 10 illustrates how the proposed method may contribute to rapid, non-invasive scoliosis screening and regular assessment of AIS in clinical practice. During an initial consultation, the portable wearable device can be quickly deployed to capture a gait sequence. The acquired motion data are first analyzed by the scoliosis screening model to identify individuals with scoliosis. For those with a scoliosis result, the data are automatically processed by the Cobb angle prediction model to provide a quantitative angle estimate, aiding in initial diagnostic triage and therapeutic planning. Following diagnosis, the system assists efficient, radiation-free longitudinal monitoring by facilitating periodic Cobb angle assessment during follow-up visits, supporting the tracking of primary curve progression or treatment response over time. Additionally, for the CNN classification model, the average inference time is approximately 0.88s per subject. For the Cobb angle prediction model, the average inference time is approximately 0.90s per subject. Both models can generate predictions within 1 s per subject on the NVIDIA RTX 5060 GPU, making it promising for scenarios requiring rapid and regular assessment.

## 6 Limitations

Several limitations of this study should be acknowledged. First, the proposed method is intended for adolescents with mild-to-moderate AIS (Cobb angle 10°–40°), the population that requires frequent assessment and prolonged monitoring, rather than for severe cases (Cobb angle >40°). A Cobb angle above 40° approaches the level at which surgery begins to be considered, and such patients are usually monitored with radiographs as part of surgical planning, which differs in both method and aim from the radiation-free assessment this study provides. Future work will extend the method to severe cases and expand the dataset across severity groups. Second, the dataset contained fewer healthy controls with a patient-to-control ratio of 104:20 (5.2:1). Accordingly, statistical comparisons were performed between healthy controls and each severity subgroup, revealing significant differences in angular features between healthy controls (20) and both the mild (n = 84) and moderate (n = 20) groups. Additionally, as shown in Table 3, the model achieved satisfactory specificity (test: 81.82%, training: 89.47%, all: 88.24%), confirming its ability to accurately identify healthy controls without bias from the class imbalance. Third, although the use of separate screening and prediction models increases system complexity, this two-stage design significantly reduces computational overhead and prevents non-scoliotic individuals from undergoing unnecessary processing by the more complex regression model, which is particularly beneficial for initial clinical deployment with limited resources. Fourth, this study focused exclusively on gait kinematics. While gait is a clinically accessible functional activity, it does not encompass other daily movements that may also reveal AIS-specific kinematics. Future work should therefore validate the proposed IMU-based system and deep learning framework across a broader set of functional tasks that may elicit more compensatory patterns. Finally, although ground truth Cobb angles were manually measured from X-ray images by experienced clinicians, introducing potential measurement error, the proposed models demonstrated competitive accuracy, offering distinct advantages of safe, portable, non-invasive and rapid assessment.

## 7 Conclusion

To enable rapid screening and longitudinal assessment of AIS, this study combined an IMU-based wearable system with deep learning models for the acquisition and analysis of kinematic angular data. Statistical analysis of angular features identified scapular motion as the most asymmetric indicator, while knee kinematics exhibited the most significant differences across severity subgroups. These findings are consistent with previous literature, supporting the validity of the proposed IMU-based wearable system. The deep learning-based classification model achieved high accuracy in discriminating between scoliosis patients and healthy individuals. The architecture of CNN-Transformer with Gaussian noise was selected as optimal for predicting Cobb angle in scoliosis patients using continuous temporal angular sequences. The integration of an IMU-based wearable and portable system with deep learning models enables a streamlined two-stage clinical workflow for AIS, offering a rapid, safe, and radiation-free approach that facilitates efficient screening, accurate initial assessment, and convenient long-term monitoring.

## Data Availability

The raw data supporting the conclusions of this article will be made available by the authors, without undue reservation.
