# Supplementary material for: Deep learning based screening and regular assessment of adolescent idiopathic scoliosis using wearable IMU sensors
Source: Front Bioeng Biotechnol. 2026 Jul 15;14:1857499. doi: 10.3389/fbioe.2026.1857499 (PMC13416696; doi:10.3389/fbioe.2026.1857499)
Supplement: Supplementary file 1 [file DataSheet1.pdf]

Supplementary Material

1 Supplementary Figures and Tables

1.1 Supplementary Tables

Supplementary Table 1. Hyperparameters of alternative Cobb angle prediction models

Hyperparameters of the CNN+Transformer (no noise)

Structure	Conv layers	Kernel size	Padding	Out-channels	Transformer		FC	Drop-out	LR	L2 regulari-zation
					d_{model}	$d_{feedforward}$				
Hyperparameter	2	(61,61)	(30,30)	(8,16)	16	64	(144, 32)	0.2	5e-5	1e-5

Hyperparameters of the CNN+LSTM

Structure	Conv layers	Kernel size	Padd-ing	Out-channels	LSTM				FC	Drop-out	LR	L2 regulari-zation
					d_{input}	d_{hidden}	*layers*	*Drop-out*				
Hyper-parameter	2	(31,31)	(15,15)	(8,16)	16	64	2	0.2	(144 ,32)	0.3	5e-5	1e-5

Hyperparameters of the CNN+GRU

Structure	Conv layers	Kernel size	Padd-ing	Out-channels	GRU				FC	Drop-out	LR	L2 regulari-zation
					d_{input}	d_{hidden}	*layers*	*Drop-out*				
Hyper-parameter	2	(61,61)	(30,30)	(32,64)	64	64	3	0.2	(576,32)	0.3	5e-5	1e-5

Hyperparameters of the CNN+FC

Structure	Conv layers	Kernel size	Padding	Out-channels	FC	Dropout	LR	L2 regularization
Hyperparameter	2	(31,31)	(15,15)	(32,64)	(576,32)	0.3	5e-5	1e-5

Hyperparameters of the Random Forest

Number of trees	Depth
3000	12

Abbreviations: Conv layers: Convolutional layers; d_{model}: Feature dimension; $d_{feedforward}$: Feed-forward hidden dimension; FC: Fully connected layer; LR: Learning rate; d_{input}: Input size; d_{hidden}: hidden dimension

1.2 Supplementary Figures

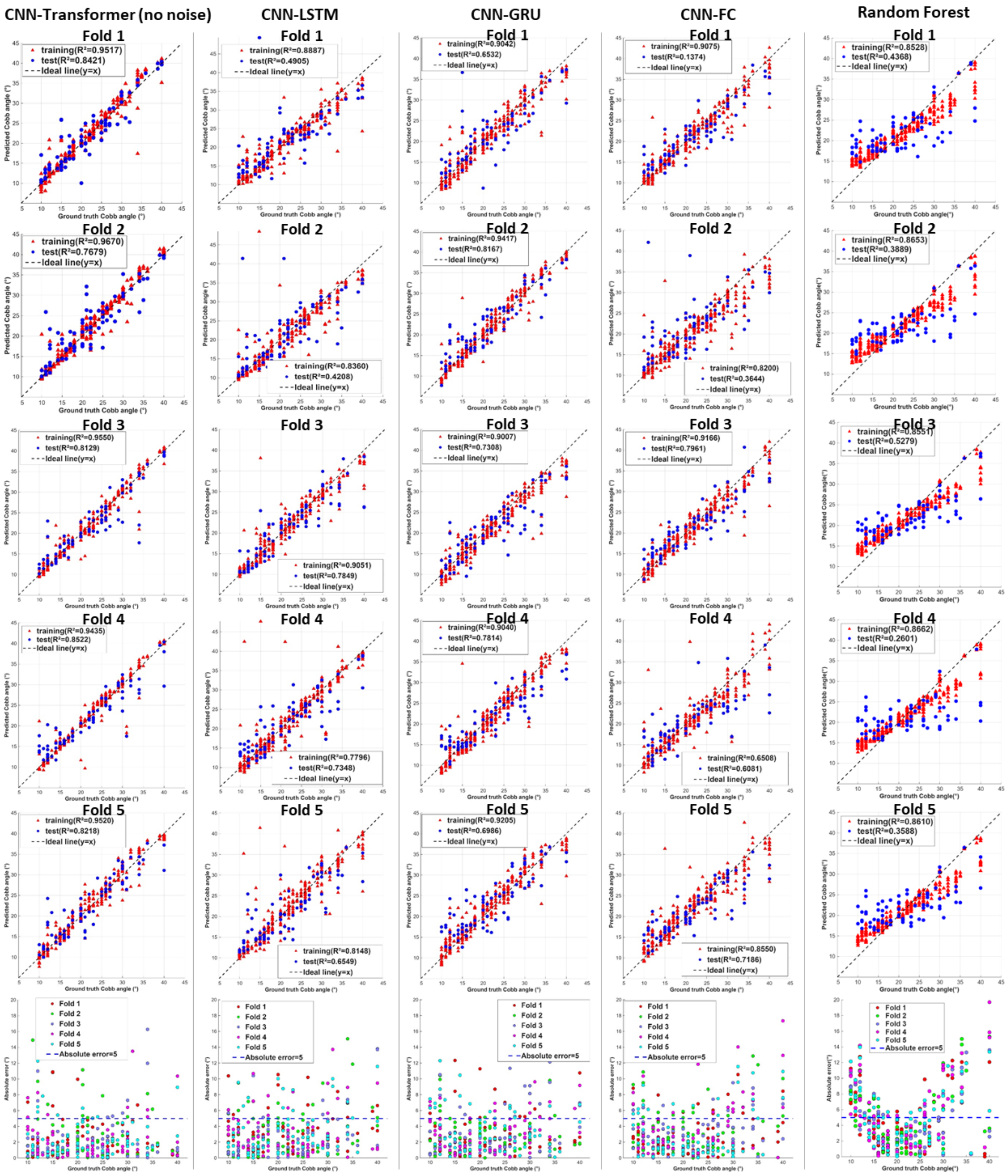

Supplementary Figure 1. Linear fitting between the predicted Cobb angles and ground truth Cobb angles in 5-fold cross-validation and the absolute errors of the five alternative models
